# Plasma methoxytyramine: clinical utility with metanephrines for diagnosis of pheochromocytoma and paraganglioma

**DOI:** 10.1530/EJE-17-0077

**Published:** 2017-05-05

**Authors:** Dipti Rao, Mirko Peitzsch, Aleksander Prejbisz, Katarzyna Hanus, Martin Fassnacht, Felix Beuschlein, Christina Brugger, Stephanie Fliedner, Katharina Langton, Christina Pamporaki, Volker Gudziol, Anthony Stell, Andrzej Januszewicz, Henri J L M Timmers, Jacques W M Lenders, Graeme Eisenhofer

**Affiliations:** 1 Department of Internal MedicineRadboud University Medical Centre, Nijmegen, The Netherlands; 2 Institute of Clinical Chemistry and Laboratory MedicineUniversity Hospital Carl Gustav Carus, Technische Universität Dresden, Dresden, Germany; 3 Department of HypertensionInstitute of Cardiology, Warsaw, Poland; 4 Department of EndocrinologyUniversity Hospital Würzburg, Würzburg, Germany; 5 Department of EndocrinologyUniversity Hospital München, München, Germany; 6 First Department of MedicineUniversity Medical Center Schleswig-Holstein, Lübeck, Germany; 7 Departments of Medicine III; 8 Departments of OtorhinolaryngologyUniversity Hospital Carl Gustav Carus, Technische Universität Dresden, Dresden, Germany; 9 Melboune eResearch GroupUniversity of Melbourne, Melbourne, Australia

## Abstract

**Context:**

Measurements of plasma methoxytyramine, the O-methylated dopamine metabolite, are useful for detecting rare dopamine-producing pheochromocytomas and paragangliomas (PPGLs) and head and neck paragangliomas (HNPGLs), but utility for screening beyond that achieved using standard measurements of normetanephrine and metanephrine is unclear.

**Objective:**

Evaluation of the additional utility of methoxytyramine compared to plasma normetanephrine and metanephrine for diagnosis of PPGLs and HNPGLs.

**Design:**

Comparative prospective study.

**Methods:**

Comparison of mass spectrometric-based measurements of plasma methoxytyramine, normetanephrine and metanephrine in 1963 patients tested for PPGLs at six tertiary medical centers according to reference intervals verified in 423 normotensive and hypertensive volunteers.

**Results:**

Of the screened patients, 213 had PPGLs and 38 HNPGLs. Using an upper cut-off of 0.10 nmol/L for methoxytyramine, 0.45 nmol/L for metanephrine and age-specific upper cut-offs for normetanephrine, diagnostic sensitivity with the addition of methoxytyramine increased from 97.2% to 98.6% for patients with PPGLs and from 22.1% to 50.0% for patients with HNPGLs, with a small decrease in specificity from 95.9% to 95.1%. Addition of methoxytyramine did not significantly alter areas under receiver operating characteristic curves for patients with PPGLs (0.984 vs 0.991), but did increase (*P* < 0.05) areas for patients with HNPGLs (0.627 vs 0.801). Addition of methoxytyramine also increased the proportion of patients with PPGLs who showed highly positive predictive elevations of multiple metabolites (70.9% vs 49.3%).

**Conclusions:**

While the benefit of additional measurements of plasma methoxytyramine for the detection of PPGLs is modest, the measurements do assist with positive confirmation of disease and are useful for the detection of HNPGLs.

## Introduction

Pheochromocytomas and paragangliomas (PPGL) are catecholamine-producing neuroendocrine tumors respectively derived from intra-adrenal and extra-adrenal chromaffin cells (1). Head and neck paragangliomas (HNPGL) in contrast do not display chromaffin cell phenotypic features or usually produce significant amounts of catecholamines (2). Catecholamines produced within chromaffin cells and their tumor derivatives are metabolized within the same cells by catechol-O-methyltransferase, a continuous process that operates independently of catecholamine secretion, explaining why the O-methylated metabolites provide superior biomarkers for PPGLs compared to their catecholamine precursors (3). While norepinephrine and epinephrine are respectively metabolized to normetanephrine and metanephrine (collectively termed metanephrines), dopamine is metabolized to methoxytyramine.

According to The Endocrine Society Clinical Practice Guidelines (4), initial biochemical testing for PPGLs should include measurements of plasma-free metanephrines or urinary fractionated metanephrines, with reference intervals and measurements for the former test preferentially carried out using blood samples collected in the supine position. Over the past decade, these tests have superseded measurements of plasma or urinary catecholamines for diagnosis of PPGLs. Nevertheless, since measurements of urinary catecholamines commonly include dopamine, it has been suggested that additional catecholamine measurements can be useful for diagnosis of dopamine-producing tumors (5). This, however, ignores the fact that most dopamine in urine is derived from renal uptake and decarboxylation of circulating L-dopa (6). Consequently, measurements of plasma methoxytyramine are superior to urinary dopamine for the detection of dopamine-producing tumors (7).

Measurements of plasma methoxytyramine have been introduced for identifying patients with metastatic PPGLs, HNPGLs and tumors due to mutations of genes encoding succinate dehydrogenase subunits (8, 9, 10). Nevertheless, the measurements are not widely offered as part of routine measurements of metanephrines. In part, this reflects difficulty in measuring the very low concentrations of free methoxytyramine in plasma, a problem now overcome by a new generation of mass spectrometers that offer higher analytical sensitivity than previously available. However, even with this problem solved, it remains unclear how much additional diagnostic utility, if any, measurements of methoxytyramine add to standard measurements of metanephrines. In particular, since plasma metanephrines offer already high diagnostic sensitivity for PPGLs, a reasonable concern of including methoxytyramine is that any additional small increase in disease detection may be entirely negated by increased numbers of false-positive results.

The above concern is compounded by reported experience of others with measurements of plasma metanephrines, where high numbers of false-positive results erode confidence that positive results can be reliably used to predict the presence of PPGLs (5, 11, 12). Problems with false-positive results can be mitigated by appropriately implemented reference intervals and attention to preanalytics, specially blood sampling in the supine position (13). For measurements of plasma methoxytyramine, it is important that patients are sampled after an overnight fast (14).

With the above considerations in mind, the present study used data collected from 1963 patients of an ongoing prospective study to assess the diagnostic utility of including measurements of plasma methoxytyramine with standard measurements of plasma-free metanephrines. An additional 423 normotensive and hypertensive volunteers were included to establish mass-spectrometric-derived reference intervals for methoxytyramine and validate reference intervals established for metanephrine and normetanephrine measured using a different analytical method (15).

## Subjects and methods

### Subjects

Subjects included 1963 patients screened for PPGLs in a multicenter prospective study (Prospective Monoamine-producing Tumor study) according to a protocol and standard-operating procedures available online (https://pmt-study.pressor.org). Patients were enrolled at 6 tertiary medical centers: (1) University Hospital Carl Gustav Carus Dresden, Germany; (2) University Medical Centre Schleswig-Holstein Lübeck, Germany; (3) University Hospital of Münich, Germany; (4) University Hospital of Würzburg, Germany; (5) Radboud University Medical Centre, Nijmegen, the Netherlands; and (6) the Institute of Cardiology, Warsaw, Poland. Enrolment was according to several criteria establishing suspicion or risk for PPGLs: (1) signs and symptoms of catecholamine excess (*n* = 794); (2) therapy-resistant hypertension (*n* = 451); (3) findings of an incidentaloma (*n* = 426); (4) hereditary risk of PPGL (*n* = 104); (5) previous history of PPGL (*n* = 178) and (6) other (*n* = 10). The reference population consisted of 423 normotensive and hypertensive volunteers (Table 1). Subjects taking tricyclic antidepressants, L-dopa or other medications known to raise plasma concentrations of O-methylated metabolites were excluded. All subjects provided written informed consent.

**Table 1:** Characteristics of reference and patient populations.

	**Group**			
	Reference	No tumors	PPGLs	HNPGLs
*n*	423	1712	213	38
Age, median and (range)	45 (18–81)	54 (10–93)	50 (11–82)	48 (26–75)
Gender, F/M	238/185	852/860	117/96	22/16

F, females; HNPGLs, head and neck paragangliomas; M, males; PPGLs, pheochromocytomas and paragangliomas.

### Tumor diagnosis and follow-up

Of the 1963 patients screened for PPGLs, tumors were confirmed in 251, including 213 with PPGLs and 38 with HNPGLs (Table 1). HNPGLs were mainly diagnosed based on testing because of hereditary risk or previous history of tumors, with routine surveillance among those at hereditary risk including imaging studies. PPGLs and HNPGLs were confirmed by pathological examination of surgically resected or biopsied tumor tissue (HNPGLs) or by diagnosis of inoperable metastatic disease based on functional imaging.

Of the patients without an initial diagnosis of PPGL, follow-up information to exclude or confirm previously undiagnosed PPGL was available in 1087 patients. Of those patients, PPGLs were excluded based on findings that all signs and symptoms were resolved (*n* = 408), an alternative diagnosis (*n* = 289), negative follow-up biochemical testing (*n* = 283) including negative results of clonidine suppression tests in patients with initial positive results (*n* = 30), alternative pathological diagnosis of a resected incidentaloma (*n* = 44), negative imaging studies (*n* = 45) or other information derived from follow-up 6 or more months after initial screening (*n* = 13). Four patients were diagnosed with PPGLs and one with a HNPGL on follow-up one or more years after initial testing.

### Blood sampling and biochemical testing

Blood samples were collected from patients and volunteers using a forearm venous cannula with subjects supine for at least 20 min before sampling. Blood samples were stored at −80°C until analysis at Dresden. Plasma-free normetanephrine, metanephrine and methoxytyramine were determined by liquid chromatography with tandem mass spectrometry (LC–MS/MS), as described elsewhere (16) and modified for measurements of methoxytyramine (17).

### Statistical analysis

Data from the reference population was used to validate upper cut-offs (UCs) previously established in a large population of over 5000 subjects for plasma metanephrines measured using a different analytical method (15). For methoxytyramine, UCs were determined using the distribution of the reference population. Differences between normo- and hypertensive reference subjects were tested by multivariate analysis after logarithmic transformation with inclusion of age and gender in the model.

True-positive results were defined in patients with tumors by plasma concentrations of any metabolite or combinations of metabolites equal to or above the UCs, whereas false-negative results were defined as concentrations of all metabolites below the UCs. False-positive results were defined in patients without tumors by plasma concentrations of any metabolite equal to or above the UCs, whereas true-negative results were defined as concentrations of all metabolites below the UCs. Diagnostic sensitivity was estimated from the percentage of true-positive results among the total of true-positive and false-negative results for patients with PPGLs. Diagnostic specificity was estimated from the percentage of true-negative results among the total of true-negative and false-positive results.

Receiver operating characteristic (ROC) curves were constructed based on multivariable logistic regression models including normetanephrine, metanephrine and methoxytyramine as characteristics, with comparisons of areas under curves (AUC) to assess differences in diagnostic test performance. Positive predictive values (posttest probability of a positive result) were calculated across prevalence rates (pretest probability) using positive likelihood ratios. Curves relating the prevalence rates and positive predictive values were constructed for different combinations of metabolites. Statistical analyses utilized the JMP statistics software package (SAS Institute Inc., Cary, NC, USA).

## Results

### Reference population and upper cut-offs

Plasma concentrations of normetanephrine did not differ between men and women, whereas concentrations of metanephrine and methoxytyramine were respectively 30% and 9% higher in men than women (Table 2). Plasma concentrations of all three metabolites showed significant positive relationships with age, the strongest for normetanephrine (*r* = 0.321, *P* < 0.0001) and weaker relationships for methoxytyramine (*r* = 0.196, *P* < 0.0001) and metanephrine (*r* = 0.130; *P* = 0.0065).

**Table 2:** Plasma concentrations (medians with ranges) for methoxytyramine, normetanephrine and metanephrine across the reference population.

	*n*	Age, median	**Methoxytyramine** (nmol/L)	**Normetanephrine** (nmol/L)	Metanephrine (nmol/L)
All subjects	423	45	0.028 (0.008–0.100)	0.340 (0.129–1.055)	0.151 (0.030–0.449)
Women	238	45	0.027 (0.008–0.078)	0.336 (0.129–0.946)	0.135 (0.030–0.449)
Men	185	46	0.029 (0.009–0.100)*	0.342 (0.156–1.055)	0.176 (0.058–0.405)**
18–29 years	83	25	0.026 (0.008–0.063)	0.281 (0.129–0.580)	0.138 (0.030–0.264)
30–39 years	65	35	0.028 (0.014–0.089)	0.301 (0.129–0.698)	0.139 (0.047–0.334)
40–49 years	116	45	0.028 (0.014–0.078)	0.320 (0.136–0.788)	0.151 (0.059–0.449)
50–59 years	100	54	0.024 (0.008–0.100)	0.368 (0.174–0.873)	0.164 (0.053–0.405)
>60 years	59	63	0.031 (0.014–0.065)	0.383 (0.156–1.055)	0.159 (0.037–0.329)
Normotensive	161	37	0.028 (0.008–0.100)^¥^	0.288 (0.129–0.788)^¥¥^	0.141 (0.042–0.449)^¥^
Hypertensive	262	50	0.029 (0.009–0.088)	0.362 (0.129–1.055)	0.161 (0.030–0.405)

* Indicates difference (*P* = 0.0031) between men and women; **indicates difference (*P* < 0.001) between men and women; ^¥^indicates difference (*P* < 0.05) between normotensive and hypertensive subjects; ^¥¥^indicates difference (*P* < 0.0001) between normotensive and hypertensive subjects.

**Figure 1:**
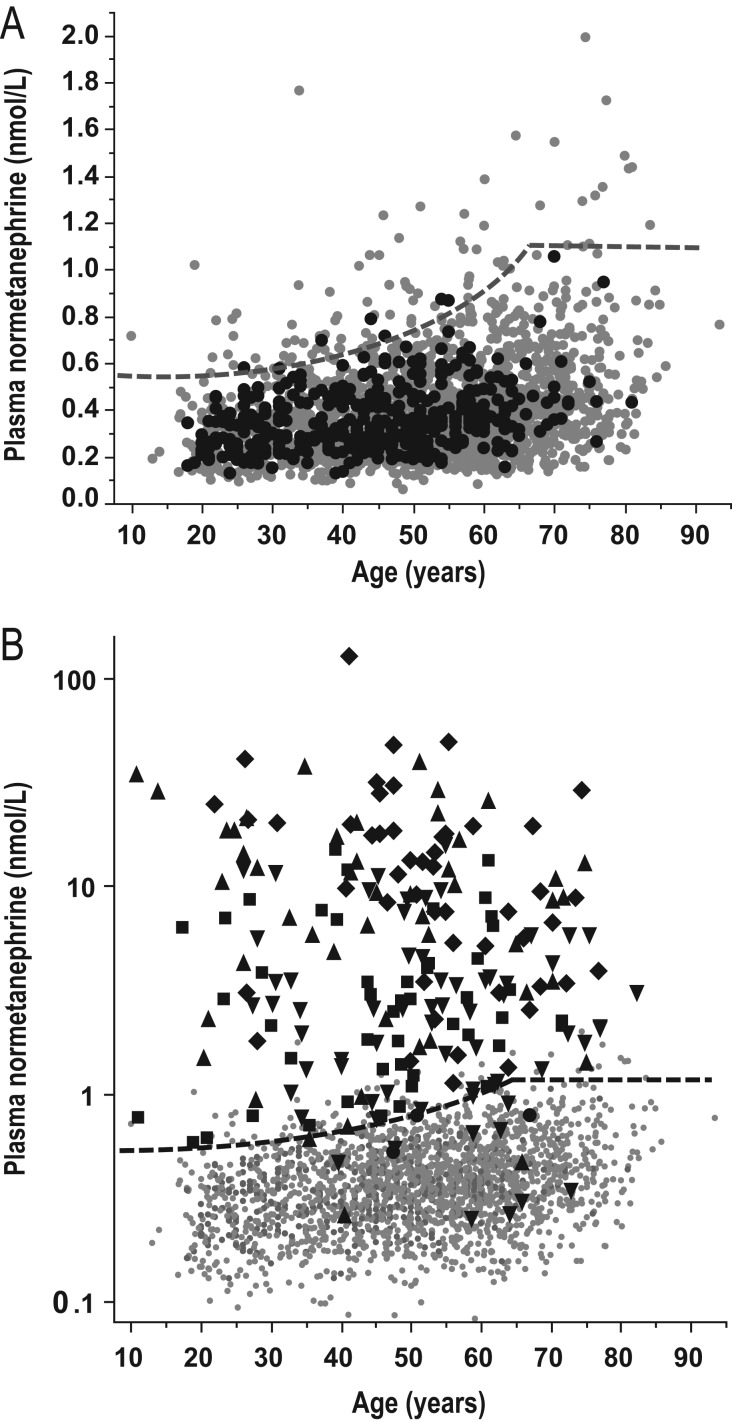
Relationships of age with plasma concentrations of normetanephrine. Panel A illustrates age relationships for subjects of the reference population (solid black dots) and patients tested for PPGLs without evidence of tumors (solid gray dots). Panel B illustrates age relationships with normetanephrine on a logarithmic scale for patients with PPGLs and positive results for normetanephrine alone (solid black square), methoxytramine or methoxytyramine and normetanephrine (solid black upward triangle), metanephrine or metanephrine and normetanephrine (solid black downward triangle), all three metabolites (solid black diamond) or no metabolites (solid gray dot) compared with patients without tumors (solid black dot). The dashed lines indicate age-specific UCs of reference intervals derived from the formula (UC nmol/L = 0.000002074 × age^3^ + 0.54) for patients 5 years to a maximum of 65 years.

Only normetanephrine, however, showed consistent age-related increases across age groups (Table 2); these differences and the relationship of normetanephrine with age showed close agreement with previously reported data in a larger reference population (15), validating the age-specific reference intervals of that population for use with LC–MS/MS-derived measurements (Fig. 1A). Thus, for plasma normetanephrine, age-specific UCs were selected according to a previously derived formula (UC nmol/L = 0.000002074 × age^3^ + 0.54; UC pg/mL = 0.0003792 × age^3^ + 98.9), but with maximum UC of 1.09 nmol/L (200 pg/mL) at age 65 (Fig. 1A), as used in the routine diagnostic service offered at Dresden since 2013. The previously established UC of 0.45 nmol/L (88 pg/mL) for metanephrine (15) was also validated by the current reference population, with only one subject showing a plasma concentration of metanephrine above that UC. The UC for methoxytyramine was selected at 0.10 nmol/L (17 pg/mL) based on the highest concentration in the reference group.

Although plasma concentrations of methoxytyramine, normetanephrine and metanephrine were all higher (*P* < 0.02) in hypertensive than normotensive groups of the reference population, these groups also differed (*P* < 0.0001) considerably in age (Table 2). Using multivariate analyses to correct for age, there were no differences in plasma concentrations of metanephrine and methoxytyramine between normotensives and hypertensives. In contrast, differences in plasma concentrations of normetanephrine persisted (*P* = 0.0044), but with correction for age, were reduced from 26% to 12% higher values in hypertensives than normotensives. The positive relationship of age with plasma normetanephrine remained highly significant (*P* < 0.0001).

### Positive test results

**Figure 2:**
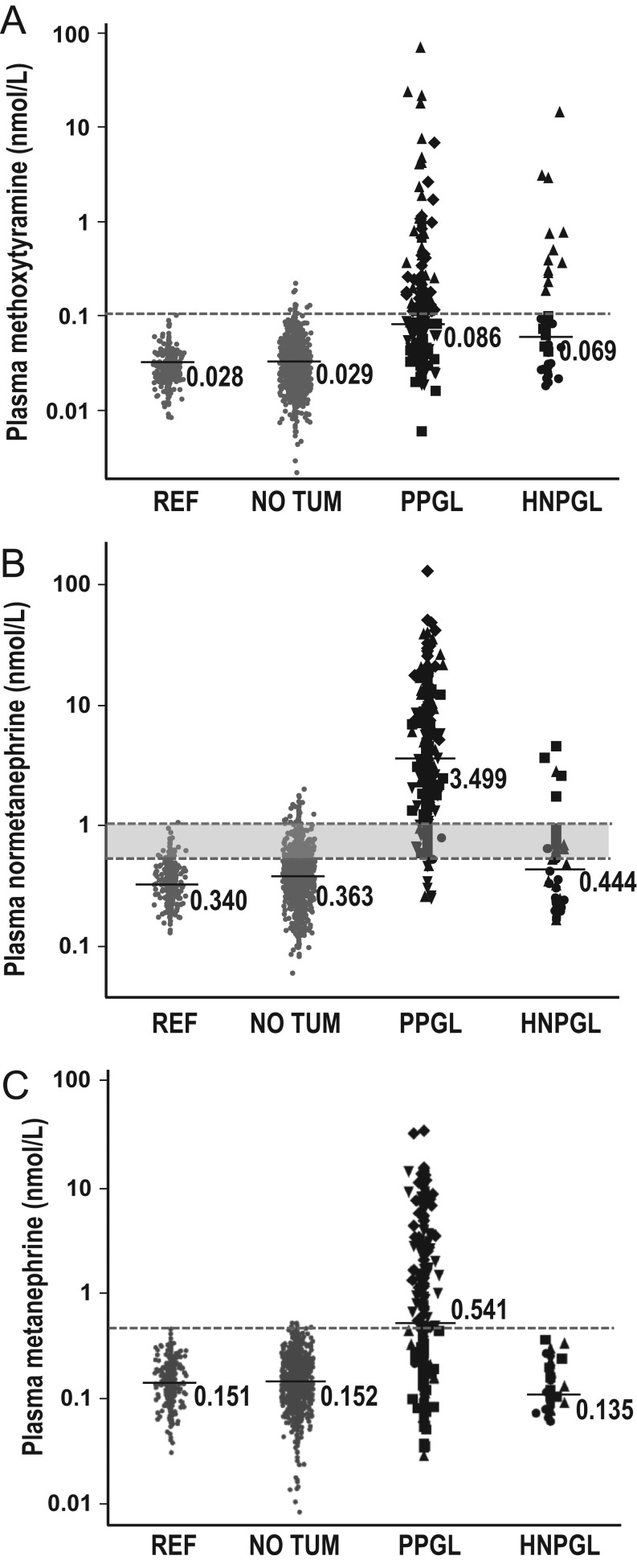
Dot plots for plasma concentrations of methoxytyramine (A), normetanephrine (B) and methanephrine (C) for the reference population (REF), patients tested for PPGLs with no evidence of tumors (NO TUM) compared to patients with PPGLs and HNPGLs. Dashed horizontal lines designate upper cut-off values of reference intervals, which for normetanephrine range from 0.55 nmol/L (100 pg/mL) in 5 -year-olds to 1.09 nmol/L (200 pg/mL) in 65 –year-olds as illustrated in Fig. 1. Median values are shown for each metabolite. Different symbols serve to illustrate subjects of the reference population or patients without tumors (solid gray dot) compared to patients with tumors and positive results for normetanephrine alone (solid black square), methoxytramine or methoxytyramine and normetanephrine (solid black upward triangle), metanephrine or metanephrine and normetanephrine (solid black downward triangle), all three metabolites (solid black diamond) or no metabolites (solid black dot).

Plasma concentrations of methoxytyramine and metanephrine were respectively increased above UCs in 45.5% and 53.5% of patients with PPGLs and 31.6% and none of the patients with HNPGLs, compared to 1.1% and 0.4% of patients without tumors (Fig. 2A and C). With application of age-specific UCs, plasma normetanephrine was increased in 93.0% of patients with PPGLs and 21.1% of patients with HNPGLs compared to 3.9% of patients without tumors (Figs 1B and 2B).

### Diagnostic sensitivity and specificity

The standard combination of normetanephrine and metanephrine yielded a diagnostic sensitivity of 97.2% for PPGLs compared to 22.1% for HNPGLs at a specificity of 95.9% (Table 3). With the addition of methoxytyramine, diagnostic specificity decreased to 95.1%, while sensitivity increased to 98.6% for the detection of PPGLs and to 50.0% for the detection of HNPGLs.

**Table 3:** Diagnostic sensitivity and specificity of measurements of plasma metanephrines with and without methoxytyramine.

	**Sensitivity** (%)		**Specificity** (%)
	PPGLs	HNPGLs	No tumor
NMN and MN	97.2 (207/213)	22.1 (8/38)	95.9 (1641/1712)
NMN, MN and MTY	98.6 (210/213)	50.0 (19/38)*	95.1 (1628/1712)

* *P* < 0.05, indicates significant difference compared to NMN and MN.

HNPGLs, head and neck paragangliomas; MN, metanephrine; MTY, methoxytyramine; NMN, normetanephrine; PPGLs, pheochromocytomas and paragangliomas.

The increased diagnostic sensitivity with inclusion of methoxytyramine reflected 3 patients with PPGLs and 11 patients with HNPGLs who showed increases in plasma concentrations of methoxytyramine above the UCs, but no increases above UCs for either normetanephrine or metanephrine. All the three patients with PPGLs and solitary increases in plasma methoxytyramine had mutations of the gene for succinate dehydrogenase subunit D and all presented with extra-adrenal paragangliomas, including one patient who also had a HNPGL.

**Figure 3:**
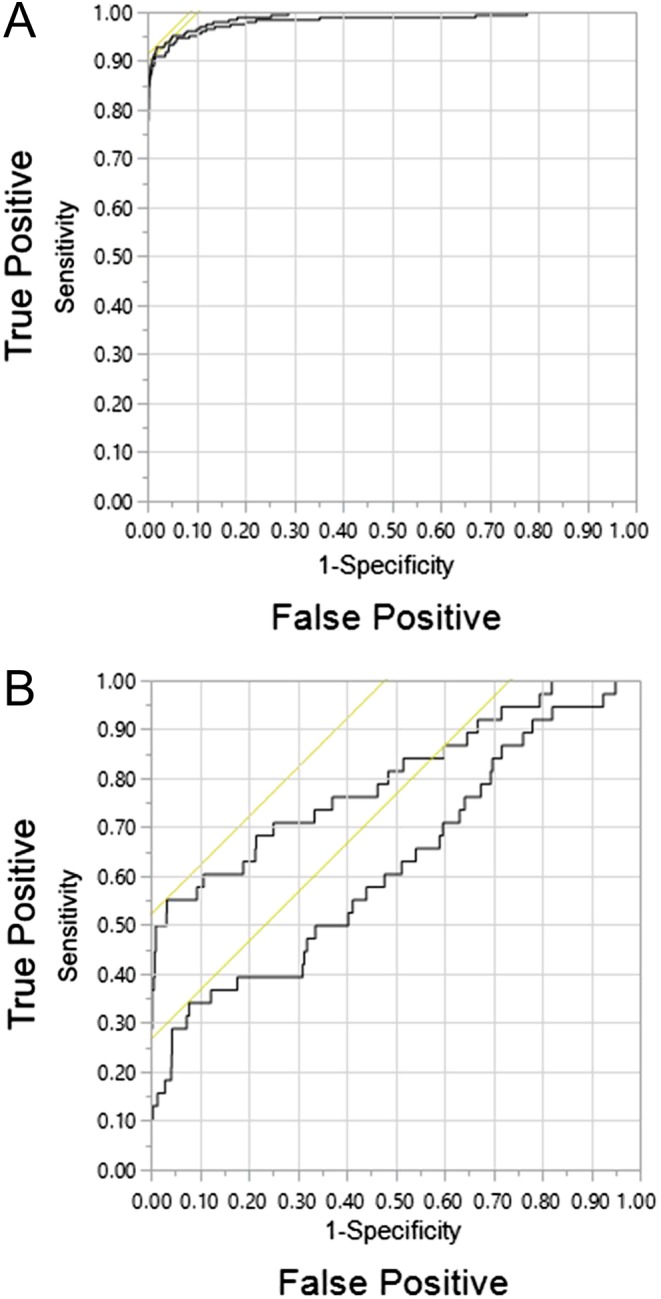
Receiver operating characteristic curves for diagnosis of PPGLs (A) and HNPGLs (B). Curves are shown for combinations of normetanephrine and metanephrine (lower curve) vs normetanephrine, metanephrine and methoxytyramine (upper curve). A full color version of this figure is available at http://dx.doi.org/10.1530/EJE-17-0077.

For diagnosis of PPGLs, areas under ROC curves did not differ with and without methoxytyramine in the test panel (0.991 vs 0.984), whereas for HNPGLs, areas under ROC curves were higher (*P* < 0.05) with than without methoxytyramine (0.801 vs 0.627) (Fig. 3).

### False-negative results

Although half of all patients with HNPGLs had concentrations of all 3 metabolites under UCs, only three of the 213 patients with PPGLs had entirely negative test results. One patient had a single 1.2 cm lymph node metastasis resected one year after biochemical testing and 3 years after resection of a 7 × 8 × 5 cm biochemically positive adrenal tumor. The two other patients had large non-functional tumors. One patient with an SDHB mutation had an 11.3 × 7.6 × 9.3 cm pelvic paraganglioma with extensive metastases that remained biochemically negative on repeated testing. The other patient had a 4.8 cm local recurrence and rapidly progressive metastatic disease at one year after the removal of a 17 × 10 × 10 cm retroperitoneal tumor.

### Follow-up diagnosis of tumors

There were five patients who were not diagnosed with tumors until follow-up, including the patient described above with the 1.2 cm lymph node metastasis and false-negative results. Another patient with a HNPGL and false-negative results showed increases of normetanephrine above age-specific UCs (1.08 nmol/L; 197 pg/mL) from 0.64 nmol/L (118 pg/mL) to 1.24 nmol/L (227 pg/mL) one year after initial testing when metastases also became evident. Two patients aged 11 and 19 years had small increases of normetanephrine (0.76 and 0.59 nmol/L; 140 and 108 pg/mL) above age-specific UCs (0.54 and 0.55 nmol/L; 99 and 101 pg/mL), but PPGLs remained undiagnosed until follow-up. The younger patient with von Hippel–Lindau syndrome showed a further increase in normetanephrine to 1.05 nmol/L (192 pg/mL) one year following initial testing, after which a clonidine test and imaging confirmed a subsequently resected adrenal pheochromocytoma. The other patient with multiple endocrine neoplasia type 2 showed respective increases of normetanephrine from 0.59 to 1.00 nmol/L (108–183 pg/mL) and of metanephrine from 0.43 to 7.79 nmol/L (85–1536 pg/mL) at 18 months after initial testing at which time a 4 cm cystic adrenal tumor was removed. The fifth patient had initially elevated normetanephrine concentrations of 1.70 nmol/L (312 pg/mL) and normal concentrations of methoxytyramine (0.05 nmol/L; 9 pg/mL) that respectively increased to 8.65 and 0.30 nmol/L (1447 and 50 pg/mL) two years later when metastatic disease was diagnosed.

### Positive predictive values

**Figure 4:**
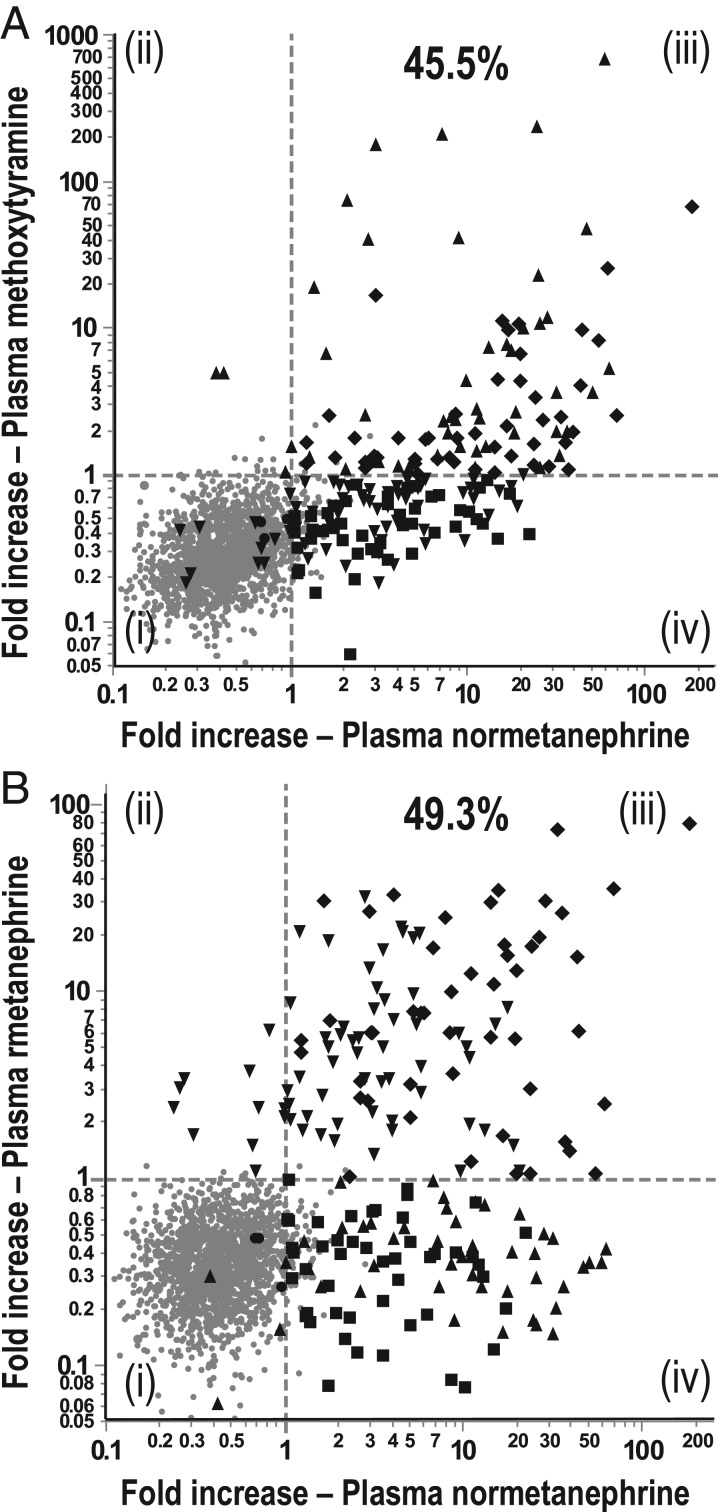
Relationships of fold increases of plasma normetanephrine above upper cut-offs vs fold increases above upper cut-offs for methoxytyramine (A) and metanephrine (B) for patient populations with and without PPGLs. Dashed vertical and horizontal lines to illustrate fold increases of 1.0 at upper cut-offs. Different symbols serve to illustrate patients without tumors (solid gray dot), patients with tumors and positive results for normetanephrine alone (solid black square), for methoxytramine or methoxytyramine and normetanephrine (solid black upward triangle), metanephrine or metanephrine and normetanephrine (solid black downward triangle), all three metabolites (solid black diamond) or no metabolites (solid black dot).

Among the patients with PPGLs, 45.5% had increases of both normetanephrine and methoxytyramine above UCs and 49.3% had increases of both normetanephrine and metanephrine above UCs compared to less than 0.3% of patients without tumors (Fig. 4). With the addition of methoxytyramine, the proportion of patients with PPGLs and combinations of positive test results increased from 49.3 to 70.9%, the latter comprising near equal proportions of patients with positive results for all three metabolites, positive results for normetanephrine and metanephrine, and positive results for normetanephrine and methoxytyramine. Respective positive results in patients without tumors were between 0.06 and 0.29%. No patient, either with or without PPGLs, had positive results for both metanephrine and methoxytyramine.

**Figure 5:**
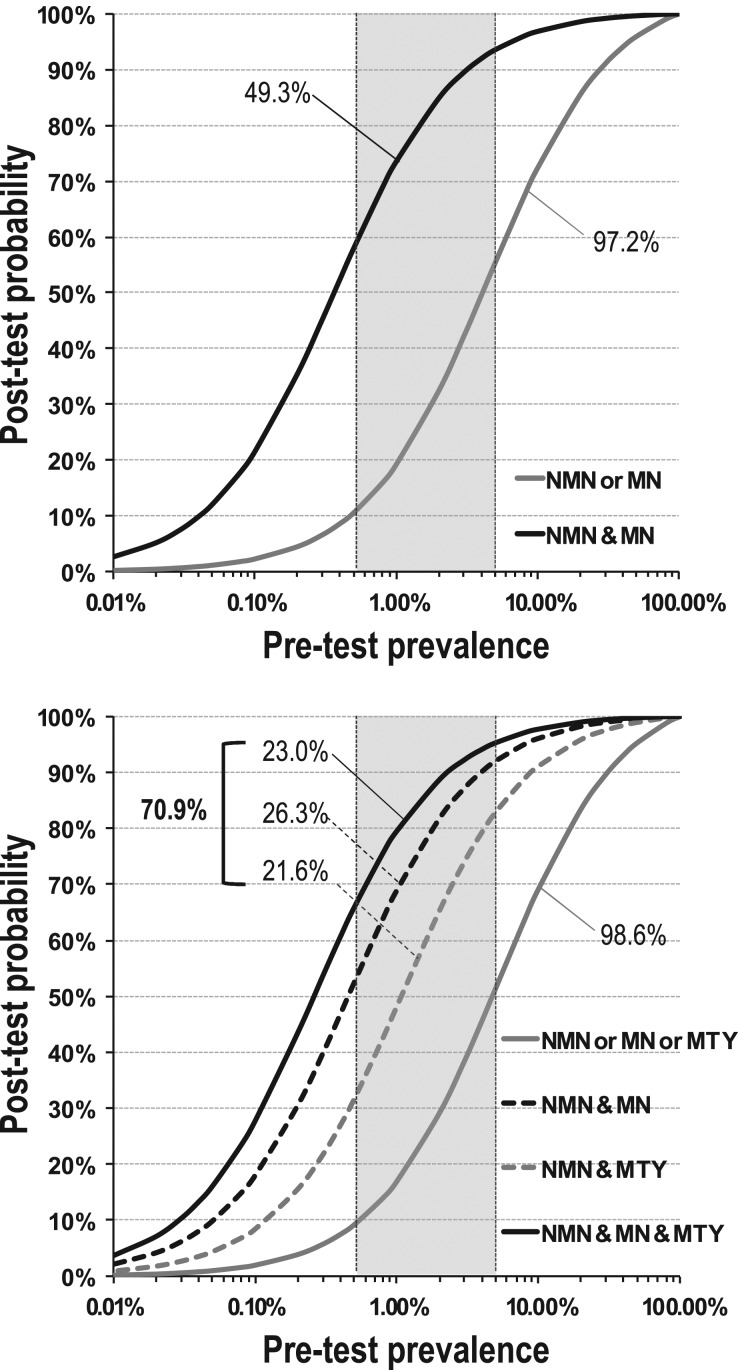
Relationships of pretest prevalence of PPGLs vs posttest probability of the tumors. Posttest probabilities are shown according to positive results for normetanephrine or metanephrine (97.2% of all patients) vs positive results for both normetanephrine and metanephrine (49.3% of all patients) in panel A. In panel B, posttest probabilities are shown according to positive results for normetanephrine or metanephrine or methoxytyramine (98.6% of all patients) vs positive results for all three metabolites (23.0% of all patients) vs positive results for both normetanephrine and metanephrine (26.3% of all patients) and vs positive results for both normetanephrine and methoxytyramine (21.6% of all patients). Shaded areas serve to illustrate posttest probabilities at common pretest prevalence of 0.5% for patients tested because of signs and symptoms to 5% in patients with incidentalomas.

Curves relating pretest prevalence to posttest positive predictive value, based on single positive results, indicated posttest probabilities ranging from 9 to 57% at pretest prevalences of 0.5–5% (Fig. 5). For positive results of both normetanephrine and metanephrine, observed in 49.3% of patients with compared to 0.18% of patients without tumors, curves were shifted to the left, ranging from 58 to 94% at pretest prevalences of 0.5–5% (Fig. 5A). For combinations of positive results for methoxytyramine, normetanephrine and metanephrine posttest probabilities at a pretest prevalence of 0.5% ranged from 32% for normetanephrine and methoxytyramine positive pairs to 52% for normetanephrine and metanephrine positive pairs, and to 66% for positive triplets of all metabolites (Fig. 5B). At a pretest prevalence of 5%, posttest probabilities were increased further ranging from 83% for normetanephrine and methoxytyramine positive pairs, 92% for normetanephrine and metanephrine positive pairs to 95% for the positive triplet combination.

Of the patients with PPGLs, 185 (86.9%) had increases of one or more of the metabolites in the triplet panel of more than 2-fold above UCs compared with only 3 patients without tumors (Fig. 3). Of these patients, 146 had combined positive results for two or more metabolites compared to a single patient without tumors, a combination indicating close to 100% probability of disease.

## Discussion

This study establishes utility of measurements of plasma methoxytyramine as an additional component of the standard panel of plasma-free metanephrines for the diagnosis of PPGLs. Although the measurements only modestly increase diagnostic sensitivity by enabling identification of rare tumors that produce solely dopamine, with appropriate reference intervals and preanalytical precautions, this detection can be achieved with a minimal loss in diagnostic specificity and no loss in diagnostic test performance as assessed by areas under ROC curves. More importantly, we show that the addition of methoxytyramine to the standard test panel improves diagnostic utility by increasing the proportion of patients with highly positive predictive test results who may benefit by a decision to immediately locate and manage the tumors rather than be subject to further follow-up biochemical testing to confirm or exclude disease. Our study also extends previous observations that measurements of methoxytyramine can be useful for identifying dopamine-producing HNPGLs (10), by significantly increasing test performance beyond that of the standard test panel.

Although there have been some previous reports on LC–MS/MS measurements of plasma metanephrines for diagnosis of PPGLs (18, 19), this is the first prospective study involving additional measurements of methoxytyramine in a large population of patients tested for the tumors. It is also the first to define and validate reference intervals for all three O-methylated metabolites that not only facilitate high diagnostic sensitivity but also enable this at a level of specificity associated with minimal false-positive results. This is important since low diagnostic specificity erodes confidence that positive results can indicate a tumor, leading to lack of motivation for follow-up (20). False-positive results, however, mainly reflect lack of adherence to preanalytical precautions, in particular sampling blood after a period of supine rest to lower sympathoneuronal release of norepinephrine and production of its O-methylated metabolite (21).

Reasonably high diagnostic specificity for measurements of normetanephrine is achieved by sampling blood in the supine position and use of age-specific UCs for supine plasma concentrations that follow the age distribution of 97.5 percentiles, doubling between 5 and 65 years. These age-specific UCs were initially established in a population of over 5000 subjects (15); they are further validated here for measurements by LC–MS/MS. Harmonization of laboratory results between methods and laboratories has been facilitated over the past decade via an international inter-laboratory quality assurance program (22), allowing for improved comparisons of results between laboratories and minimized requirements for validation of transferred reference interval data.

For measurements of metanephrine and methoxytyramine, which are of secondary importance to normetanephrine for diagnosis of PPGLs, high diagnostic sensitivity can be maintained with minimal loss of specificity by establishing UCs using 99.5 percentiles or ranges in reference populations rather than commonly employed 97.5 percentiles. For measured concentrations of plasma methoxytyramine, which can be increased by the presence of dietary amine precursors (14), it is also critical that blood sampling is performed after an overnight fast (21). With these precautions and UCs in place, the present report establishes minimal proportions of false-positive results for all metabolites and even lower proportions of false-positives for 2 or more metabolites in the panel.

The rare nature of positive results involving increased plasma concentrations of two or more metabolites in patients without compared to those with PPGLs means that such findings are strongly predictive of a catecholamine-producing tumor. This is useful since at low pretest prevalences, even with diagnostic specificity approaching 95%, posttest predictive values of a positive result for a single metabolite can be insufficient for reliable confirmation of a tumor. Although pretest prevalences of PPGLs can run up to 5% in patients with adrenal incidentalomas (23), among unselected patients screened for PPGLs pretest prevalences range from 0.8% to 1.6% (24, 25, 26), which is in line with lower prevalences of PPGLs among hypertensives of up to 0.6% (27). As we show here, at such prevalences posttest probabilities may not reach more than 10% for single positive results.

The Endocrine Society Guidelines on PPGLs include recommendations that all positive biochemical results should be followed up, while imaging studies to locate tumors should not be initiated until there is clear biochemical evidence of a PPGL (4). This raises the question about what constitutes clear biochemical evidence. As outlined in those guidelines, increases of both normetanephrine and metanephrine provide such evidence. As we now outline here, the addition of methoxytyramine to the test panel increases to over 70% the proportion of patients with highly predictive positive results for multiple metabolites. For those patients, there should be no need to embark on confirmatory biochemical tests. Rather the immediate task is to locate the tumor.

Addition of methoxytyramine to standard tests of normetanephrine and metanephrine has already established utility, beyond screening for PPGLs, by pointing to possible metastatic disease (9) or the presence of mutations in genes for succinate dehydrogenase (8). While not the focus of the present study, this additional utility of methoxytyramine is also important when considering the addition of these measurements to the standard test panel routinely offered for diagnosis of PPGLs and particularly when there is additional risk of HNPGLs.

One limitation of our study relates to the follow-up of patients without a diagnosis of PPGLs to further exclude or confirm disease, this achieved in 63.4% of patients, with exclusion of PPGLs in 99.5% of those cases. Of the five patients in whom tumors were found on follow-up, two were patients with false-negative results including one with an HNPGL and another with a small lymph node metastasis secondary to a previously resected adrenal pheochromocytoma. All the three other patients with PPGLs discovered on follow-up showed initial small increases in normetanephrine above UCs, illustrating the importance of not to ignore any patient with positive biochemical test results.

Although false-negative results are common among patients with HNPGLs (10), in this study involving 50% of patients, we confirm that false-negative results are rare for patients with PPGLs when employing measurements of plasma-free metanephrines and methoxytyramine. As shown here and elsewhere (28, 29), such negative results may be encountered in patients with small tumors or metastatic lesions (<1.5 cm) as well as in patients with non-functional PPGLs that do not synthesize, store or metabolize catecholamines. Non-functional tumors may reach a large size before diagnosis (29), as also indicated by the two cases in this series. Apart from these rare tumors, negative test results for plasma metanephrines and methoxytyramine reliably exclude all but the smallest of catecholamine-producing PPGLs.

In summary, while additional measurements of plasma methoxytyramine only modestly improve detection of PPGLs above that achieved using standard measurements of normetanephrine and metanephrine, the measurements are useful for the detection of HNPGLs. More importantly, inclusion of methoxytyramine enables more accurate discrimination of true-positive from false-positive results. Specifically, even at pretest prevalences as low as 0.5%, combinations of positive results for any two or more of the three metabolites carry high positive predictive value that along with increases of 2-fold or more above UCs indicate close to 100% probability of tumors in nearly 70% of patients with PPGLs. It is however important to appreciate that these conclusions are only valid with accurate measurements of the metabolites, appropriate attention to preanalytics and correctly established reference intervals.

## Declaration of interest

The authors declare that there is no conflict of interest that could be perceived as prejudicing the impartiality of this study.

## Funding

This work was supported by the Deutsche Forschungsgemeinschaft (EI855/1/2) and the European Union Horizon 2020 Program (ENS@T-HT – #633983-2).

## References

[1] Lenders JW Eisenhofer G Mannelli M Pacak K. Phaeochromocytoma. Lancet 2005 366 665–675. (10.1016/S0140-6736(05)67139-5) 16112304

[2] Tischler AS. Pheochromocytoma and extra-adrenal paraganglioma: updates. Archives of Pathology and Laboratory Medicine 2008 132 1272–1284. (10.1043/1543-2165(2008)132[1272:PAEPU]2.0.CO;2) 18684026

[3] Eisenhofer G Huynh TT Hiroi M Pacak K. Understanding catecholamine metabolism as a guide to the biochemical diagnosis of pheochromocytoma. Reviews in Endocrine and Metabolic Disorders 2001 2 297–311. (10.1023/A:1011572617314) 11708294

[4] Lenders JWM Duh QY Eisenhofer G Gimenez-Roqueplo AP Grebe SK Murad MH Naruse M Pacak K Young WF Jr Endocrine Society Pheochromocytoma and paraganglioma: an Endocrine Society Clinical Practice Guideline. Journal of Clinical Endocrinology and Metabolism 2014 99 1915–1942. (10.1210/jc.2014-1498) 24893135

[5] Young WF Jr Clinical practice. The incidentally discovered adrenal mass. New England Journal of Medicine 2007 356 601–610. (10.1056/NEJMcp065470) 17287480

[6] Brown MJ Allison DJ. Renal conversion of plasma DOPA to urine dopamine. British Journal of Clinical Pharmacology 1981 12 251–253. (10.1111/j.1365-2125.1981.tb01210.x) 6796105 PMC1401852

[7] Eisenhofer G Goldstein DS Sullivan P Csako G Brouwers FM Lai EW Adams KT Pacak K. Biochemical and clinical manifestations of dopamine-producing paragangliomas: utility of plasma methoxytyramine. Journal of Clinical Endocrinology and Metabolism 2005 90 2068–2075. (10.1210/jc.2004-2025) 15644397

[8] Eisenhofer G Lenders JW Timmers H Mannelli M Grebe SK Hofbauer LC Bornstein SR Tiebel O Adams K Bratslavsky G Measurements of plasma methoxytyramine, normetanephrine, and metanephrine as discriminators of different hereditary forms of pheochromocytoma. Clinical Chemistry 2011 57 411–420. (10.1373/clinchem.2010.153320) 21262951 PMC3164998

[9] Eisenhofer G Lenders JW Siegert G Bornstein SR Friberg P Milosevic D Mannelli M Linehan WM Adams K Timmers HJ Plasma methoxytyramine: a novel biomarker of metastatic pheochromocytoma and paraganglioma in relation to established risk factors of tumour size, location and SDHB mutation status. European Journal of Cancer 2012 48 1739–1749. (10.1016/j.ejca.2011.07.016) 22036874 PMC3372624

[10] van Duinen N Corssmit EP de Jong WH Brookman D Kema IP Romijn JA. Plasma levels of free metanephrines and 3-methoxytyramine indicate a higher number of biochemically active HNPGL than 24-h urinary excretion rates of catecholamines and metabolites. European Journal of Endocrinology 2013 169 377–382. (10.1530/EJE-13-0529) 23832865

[11] Sawka AM Prebtani AP Thabane L Gafni A Levine M Young WF Jr A systematic review of the literature examining the diagnostic efficacy of measurement of fractionated plasma free metanephrines in the biochemical diagnosis of pheochromocytoma. BMC Endocrine Disorders 2004 4 2 (10.1186/1472-6823-4-2) 15225350 PMC459231

[12] Yu R Wei M. False positive test results for pheochromocytoma from 2000 to 2008. Experimental and Clinical Endocrinology and Diabetes 2010 118 577–585. (10.1055/s-0029-1237699) 19998239

[13] Eisenhofer G Peitzsch M. Laboratory evaluation of pheochromocytoma and paraganglioma. Clinical Chemistry 2014 60 1486–1499. (10.1373/clinchem.2014.224832) 25332315

[14] de Jong WH Eisenhofer G Post WJ Muskiet FA de Vries EG Kema IP. Dietary influences on plasma and urinary metanephrines: implications for diagnosis of catecholamine-producing tumors. Journal of Clinical Endocrinology and Metabolism 2009 94 2841–2849. (10.1210/jc.2009-0303) 19567530

[15] Eisenhofer G Lattke P Herberg M Siegert G Qin N Darr R Hoyer J Villringer A Prejbisz A Januszewicz A Reference intervals for plasma free metanephrines with an age adjustment for normetanephrine for optimized laboratory testing of phaeochromocytoma. Annals of Clinical Biochemistry 2013 50 62–69. (10.1258/acb.2012.012066) 23065528 PMC4714582

[16] Peitzsch M Prejbisz A Kroiss M Beuschlein F Arlt W Januszewicz A Siegert G Eisenhofer G. Analysis of plasma 3-methoxytyramine, normetanephrine and metanephrine by ultraperformance liquid chromatography-tandem mass spectrometry: utility for diagnosis of dopamine-producing metastatic phaeochromocytoma. Annals of Clinical Biochemistry 2013 50 147–155. (10.1258/acb.2012.012112) 23512172

[17] Peitzsch M Adaway JE Eisenhofer G. Interference from 3-O-methyldopa with ultra-high performance LC-MS/MS measurements of plasma metanephrines: chromatographic separation remains important. Clinical Chemistry 2015 61 993–996. (10.1373/clinchem.2015.239962) 25931454

[18] Peaston RT Graham KS Chambers E van der Molen JC Ball S. Performance of plasma free metanephrines measured by liquid chromatography-tandem mass spectrometry in the diagnosis of pheochromocytoma. Clinica Chimica Acta 2010 411 546–552. (10.1016/j.cca.2010.01.012) 20083099

[19] Kim HJ Lee JI Cho YY Lee SY Kim JH Jung BC Kim SW Chung JH Min YK Lee MS Diagnostic accuracy of plasma free metanephrines in a seated position compared with 24-hour urinary metanephrines in the investigation of pheochromocytoma. Endocrine Journal 2015 62 243–250. (10.1507/endocrj.EJ14-0384) 25476661

[20] Anas SS Vasikaran SD. An audit of management of patients with borderline increased plasma-free metanephrines. Annals of Clinical Biochemistry 2010 47 554–558. (10.1258/acb.2010.010131) 20926470

[21] Darr R Pamporaki C Peitzsch M Miehle K Prejbisz A Peczkowska M Weismann D Beuschlein F Sinnott R Bornstein SR Biochemical diagnosis of phaeochromocytoma using plasma-free normetanephrine, metanephrine and methoxytyramine: importance of supine sampling under fasting conditions. Clinical Endocrinology 2014 80 478–486. (10.1111/cen.12327) 24102244

[22] Pillai D Callen S. Pilot quality assurance programme for plasma metanephrines. Annals of Clinical Biochemistry 2010 47 137–142. (10.1258/acb.2009.009153) 20144968

[23] Mantero F Terzolo M Arnaldi G Osella G Masini AM Alì A Giovagnetti M Opocher G Angeli A. survey on adrenal incidentaloma in Italy. Study group on adrenal tumors of the Italian Society of Endocrinology. Journal of Clinical Endocrinology and Metabolism 2000 85 637–644. (10.1210/jc.85.2.637) 10690869

[24] Hernandez FC Sanchez M Alvarez A Díaz J Pascual R Pérez M Tovar I Martínez P. A five-year report on experience in the detection of pheochromocytoma. Clinical Biochemistry 2000 33 649–655. (10.1016/s0009-9120(00)00172-7) 11166012

[25] Vaclavik J Stejskal D Lacnak B Lazárová M Jedelský L Kadalová L Janosová M Frysák Z Vlcek P. Free plasma metanephrines as a screening test for pheochromocytoma in low-risk patients. Journal of Hypertension 2007 25 1427–1431. (10.1097/HJH.0b013e32813aeb5a) 17563565

[26] Brain KL Kay J Shine B. Measurement of urinary metanephrines to screen for pheochromocytoma in an unselected hospital referral population. Clinical Chemistry 2006 52 2060–2064. (10.1373/clinchem.2006.070805) 16990424 PMC2640466

[27] Omura M Saito J Yamaguchi K Kakuta Y Nishikawa T. Prospective study on the prevalence of secondary hypertension among hypertensive patients visiting a general outpatient clinic in Japan. Hypertension Research 2004 27 193–202. (10.1291/hypres.27.193) 15080378

[28] Lenders JW Pacak K Walther MM Linehan WM Mannelli M Friberg P Keiser HR Goldstein DS Eisenhofer G. Biochemical diagnosis of pheochromocytoma: which test is best? Journal of the American Medical Association 2002 287 1427–1434. 11903030 10.1001/jama.287.11.1427

[29] Timmers HJ Pacak K Huynh TT Abu-Asab M Tsokos M Merino MJ Baysal BE Adams KT Eisenhofer G. Biochemically silent abdominal paragangliomas in patients with mutations in the succinate dehydrogenase subunit B gene. Journal of Clinical Endocrinology and Metabolism 2008 93 4826–4832. (10.1210/jc.2008-1093) 18840642 PMC2626451

